# CB-RAF600E-1 exerts efficacy in vemurafenib-resistant and non-resistant-melanoma cells via dual inhibition of RAS/RAF/MEK/ERK and PI3K/Akt signaling pathways

**DOI:** 10.1016/j.sjbs.2022.103285

**Published:** 2022-04-23

**Authors:** Mesfer Al Shahrani, Prasanna Rajagopalan, Mohammad Abohassan, Mohammad Alshahrani, Yasser Alraey

**Affiliations:** a Department of Clinical Laboratory Sciences, College of Applied Medical Sciences, King Khalid University, Abha, Saudi Arabia; b Central Research Laboratory, College of Applied Medical Sciences, King Khalid University, Abha, Saudi Arabia

**Keywords:** High throughput, BRAFV600E, ERK, Akt, Melanoma, Vemurafenib, Resistance

## Abstract

**Background and Aim:**

Predicting novel dual inhibitors to combat adverse effects such as the development of resistance to vemurafenib in melanoma treatment due to the reactivation of MAPK and PI3K/AKT signaling pathways is studied to help in reversal of cancer symptoms.

Reversal of cancer symptoms in melanoma associated with vemurafenib resistance is driven by reactivation of MAPK and PI3K/Akt signaling pathways. Novel dual inhibitors targeting these proteins would be beneficial to combat resistance.

**Methods:**

High-throughput virtual screening of the ChemBridge library against B-RAFV600E and Akt was performed using an automated protocol with the AutoDock VINA program. Luminescence and time-resolved fluorescence kits were used to measure enzyme activities. The MTT assay was used to determine proliferation in normal and vemurafenib-resistant A375 cells. Flow cytometry was used to examine apoptosis, cell cycle, and phosphorylation of ERK/Akt signaling pathway.

**Results:**

High-throughput screening from the ChemBridge library identified 15 compounds with high binding energy towards B-RAFV600E; among these, CB-RAF600E-1 had the highest ΔG_binding_ score −11.9 kcal/mol. The compound also had a high affinity towards Akt, with a ΔG_binding_ score of −11.5 kcal/mol. CB-RAF600E-1 dose-dependently inhibited both B-RAFV600E and Akt with IC_50_ values of 635 nM and 154.3 nM, respectively. The compound effectively controlled the proliferations of normal and vemurafenib-resistant A375 cells, with GI_50_ values of 222.3 nM and 230.5 nM, respectively. A dose-dependent increase in the sub G_0_/G_1_ phase of the cell cycle and total apoptosis was observed following compound treatment in both normal and vemurafenib-resistant melanoma cells. Treatment with CB-RAF600E-1 decreased the pERK/pAkt dual-positive populations in normal and vemurafenib-resistant A375 cells.

**Conclusion:**

CB-RAF600E-1, identified as a novel dual inhibitor effective against normal and vemurafenib-resistant melanoma cells, requires further attention for development as an effective chemotherapeutic agent for melanoma management.

## 1 Introduction

Melanoma, an aggressive solid tumor of the melanocytes, has reached alarming proportions in the population. Despite regular treatments, surgical interventions, it escalates to stage IV in a majority of patients due to metastasis within a short span of time and is responsible for 95% of fatality.

Melanoma is currently the fifth most common type of tumor, with an alarming increase in the incidence rate compared to other solid malignancies (Jemal et al., 2010). Although melanoma can be successfully managed with surgical restrictions, the majority of patients are prone to develop disseminated disease associated with distant metastasis. Despite regular treatment, advancement of melanoma to stage IV is fatal for over 95% of the patients within the first to fifth year of disease progression (Sullivan and Flaherty, 2011).

The mitogen-activated protein kinase (MAPK) pathway uses serine/threonine RAF kinases as survival signals for cell proliferation (Peyssonnaux and Eychène, 2001). This pathway uses the RAS-RAF-MEK cascade (also referred to as the ERK signal), which is widely activated in nearly 30% of all human tumors (Wellbrock et al., 2004). Of all members of the ERK family, B-RAF is a therapeutic target in many cancer types (Arkenau et al., 2011). Mutations in B-RAF are widely reported in an array of cancer types, including melanoma, thyroid, colorectal, non-small-cell lung, and ovarian cancers, gliomas, and leukemia (Tang et al., 2015). Studies indicate that over 40% of melanoma patients bear a mutated B-RAF (Leicht et al., 2007, Siroy et al., 2015). Among the assessed mutation variation, V600E accounts for nearly 90% of the B-RAF mutations, imparting a negative charge on the kinase domain of B-RAF, thereby resulting in constitutive MAPK signaling for tumor progression (Davies et al., 2002). Consequently, B-RAFV600-E has become an attractive target for melanoma control.

Advancements in precision medicine have led to high-throughput screening of many small molecules for selective inhibition of B-RAFV600-E with high efficacy and selectivity (Pejin et al., 2013). Of these, vemurafenib was the first highly potent inhibitor of phase I clinical trials with the highest clinical success rate (Flaherty et al., 2010). Vemurafenib treatment reduced ERK phosphorylation (pERK) in B-RAFV600-E mutated melanoma cells to exhibit an excellent therapeutic clinical response (Bollag et al., 2010). However, the positive impact of the treatment was reversed due to acquired drug resistance. Hyper- or over-activation of RTKs, reactivation of MAPK signals, and hyper-activation of the PI3K/Akt pathways have been reported in vemurafenib-resistant patients. Such alterations of these key pathways impact the tumor microenvironment, which tantamount to negating the vemurafenib treatment. In order to combat vemurafenib resistance, novel small-molecule combinations for different targets would be beneficial. However, the use of multiple numbers and/or doses of drugs can result in adverse side effects. As an alternative, single small molecules with dual inhibition potential would be more rewarding in terms of efficacy and least toxicity. This approach is also beneficial to combat resistant melanoma cells as they target multiple proteins. Thus, this study focuses on the screening of novel B-RAFV600-E/Akt dual inhibitors using a computational approach and validating the lead compound against normal and vemurafenib-resistant melanoma cells.

## 2 Materials & methods

### 2.1 Materials

Reagents and chemicals were purchased from Sigma-Aldrich (St. Louis, MO, USA). The A375 cell line was obtained from the American Type Culture Collection (ATCC, Rockville, MD, USA). Annexin V, cell cycle assay reagents, and Flow Collect™ PI3K/MAPK Dual Pathway Activation and Cancer Marker Detection Kit were purchased from Merck Millipore (Burlington, MA, USA). B-RAFV600E kinase enzyme assay kits were purchased from BPS Bioscience (San Diego, CA, USA). The Akt enzyme kit was purchased from Invitrogen (USA).

### 2.2 Methods

#### 2.2.1 Structure modelling and small-molecule database preparation

The three-dimensional crystal structures of B-RAF-V600E (PDB ID 4MNF) and Akt (PDB ID 6HHG) were retrieved from the Protein Databank. Prior to the docking protocol, structures were prepared using a receptor preparation script from AutoDockTools. Structures of known B-RAFV600E inhibitors were retrieved from the PubChem database in SDF format. The small-molecule database (KINAcore and KINAset) was retrieved from the ChemBridge database in SDF format. All SDF-format ligands were converted to SYBYL-TRIPOS (mol2) format using the BIOVIA-Discovery Studio Visualizer. Subsequently, the mol2 files were converted to AutoDock VINA format using a ligand preparation script from AutoDockTools.

#### 2.2.2 High-throughput virtual docking

High-throughput virtual screening of the ChemBridge library against B-RAF-V600E was performed using the automated protocol developed by SiBIOLEAD. Briefly, a docking box was generated based on the information gained from the B-RAFV600E structure complexed with sorafenib (1UWH) by selecting two amino acid residues on either side of the active site. The AutoDock VINA program was used for the high-throughput docking analysis. To accelerate the screening process, the exhaustiveness was set to two. The small molecules were ranked based on their docking scores.

#### 2.2.3 Standard protein–ligand docking

Out of the 23,365 compounds, 15 lead compounds identified from the high-throughput virtual screening were again docked with B-RAF-V600E using the same docking box information and the AutoDock VINA package with standard docking mode (i.e., exhaustiveness = 8). The top lead compound was ranked based on its docking score. Protein-ligand interactions were analyzed using the PLIP protein–ligand analysis package. A similar docking protocol was performed with Akt kinase as the top lead compound.

#### 2.2.4 *In vitro* kinase inhibition assays

B-RAF-V600E enzyme assay was performed using a luminescence-based kinase assay kit (BPS Bioscience) according to the manufacturer’s instructions. Briefly, a master mix with a final volume of 25 µL containing kinase buffer, 500 µM ATP, RAF substrate, and water was added to each well of a 96-well white bottom plate. DMSO (5 μL) or various concentrations of CB-RAF600E-1 (0.1 nM to 10,000 nM in log scales) were added to the wells, and 2 ng/µL B-RAF-V600E was added to initiate the reaction. Suitable blanks contained 5 µL of kinase buffer without the enzyme. The plate was incubated at 30 °C for 45 min in the dark. Kinase-Glo Max reagent (50 µL) was added to each well and incubated for 15 min at 25 C^o^. Luminescence was measured using a FLUOstar Omega microplate reader (BMG Labtech, Ortenberg, Germany). The Akt inhibition assay was performed with 0.1 nM to 10,000 nM in log scale concentrations of compound using the Z'-LYTE™ kit (Invitrogen) according to the manufacturer’s instructions. The ratio of fluorescence emission at 520 nm to coumarin emission at 445 nm was used to quantify the reaction progress. Fluorescence energy transfer (FRET) was measured using a FLUOstar Omega microplate reader (BMG Labtech). GraphPad Prism was used to calculate IC_50_ values for both enzyme assays.

#### 2.2.5 Cell culture and resistance establishment

A375 cells were grown as per standard protocols in regular DMEM supplemented with 10% fetal bovine serum, 100 U/mL penicillin, and 100 U/mL streptomycin. Cells were maintained at 37 °C and 5% CO_2_ with regular trypsinization and re-seeding. To acquire resistance, B-RAF V600E inhibitor, 0.05 µM vemurafenib was added in the first week, with double the concentration of vemurafenib every subsequent week. The sensitivity of the cells to vemurafenib was checked every alternate week, and the final concentration of vemurafenib used for the resistant cell line was 3.5 µM. The normal cell line and vemurafenib cell line were named A375-N and A-375-R, respectively. Resistance to vemurafenib was evaluated based on the change in GI_50_ response to A375-R compared to A375-N, and the resistance was confirmed with every set of passages, wherever the A375-R cell line was used in subsequent assays.

#### 2.2.6 Cell proliferation assays

Cell proliferation was assessed using the MTT assay, as previously described (Prasanna and Harish, 2010). A375-N or A375-R cells (5 × 10^3^ cells/well) were grown in 96-well tissue culture plates in regular growth medium. Cells were treated with the respective concentrations of vemurafenib or CB-RAF600E-1 for 48 h. After removing the medium, cells were treated with 100 μL MTT (1 mg/mL) and further incubated for 4 h. Formazan products were dissolved in 200 µL of DMSO, and the absorbance at 560 nm was measured. Percent inhibition was calculated using GraphPad Prism 6.0 to determine GI_50_ values.

#### 2.2.7 Cell cycle analysis

The assay was performed using a cell cycle assay kit, according to the manufacturer’s instructions. A375-N or A375-R cells at a density of 0.5 × 10^6^ cells/well were seeded in a 6-well plate and incubated for 24 h. After adding 100 nM or 200 nM CB-RAF600E-1, the cells were incubated for 72 h. After washing twice with sterile phosphate-buffered saline (PBS), cells were treated with 50 μL cell cycle assay reagent, incubated in the dark for 15 min, washed twice with wash buffer, and resuspended in HBSS buffer. Ten thousand events were acquired on a Guava easyCyte flow cytometer, and the data were analyzed with ExpressPro Software from Millipore. The percentage of the cell population in the sub G_0_/G_1_ phase is presented.

#### 2.2.8 Apoptosis analysis by annexin V assay

Apoptosis in normal and resistant A375 cells was quantified using an annexin V detection kit according to the manufacturer’s instructions. A375-N or A375-R cells (0.5 × 10^6^) were grown in 6-well plates and treated with the desired concentrations of CB-RAF600E-1, followed by incubation in 5% CO_2_ at 37 °C for 48 h. Following incubation, cells were harvested, washed with kit buffer, and incubated with 0.25 µg/mL annexin V reagent for 15 min in the dark. After washing twice, cells were re-suspended in a kit buffer containing 0.5 µg/mL propidium iodide. Ten thousand events were acquired on a Guava easyCyte flow cytometer. Data analysis was carried out using InCyte software to differentiate between healthy and apoptotic cells (early and late apoptosis) and presented using GraphPad Prism software (version 6.0; La Jolla, CA, USA).

#### 2.2.9 pERK/pAkt dual inhibition assay by flow cytometry

A375-N or A375-R cells were pre-treated with 200 nM CB-RAF600E-1 and incubated for 4 h in a 5% CO_2_ incubator at 37 °C, with suitable untreated and induction controls. After incubation, the induction control and drug-treated cells alone were induced with 50 ng/mL PMA for 5 min. All cells were washed twice with sterile PBS and resuspended in the HBSS buffer. A375-N or A375-R cells were treated with 0.50 μg/mL pERK-PE/pAkt-Alexa 488 dual antibody and further incubated for 30 min in the dark. Cells were washed twice with PBS and resuspended in the HBSS buffer. Ten thousand events were acquired using the Guava easyCyte flow cytometer, and the data were analyzed using ExpressPro Software. The percentage of positive cells in each quadrant is shown.

#### 2.2.10 Statistical analysis

Statistical analyses were performed using GraphPad Prism 6.0 (La Jolla, USA). The experiments were performed in triplicate and all data represented the mean ± S.D. GI_50_, IC_50_ values were calculated using a non-linear regression fit model with variable slope and plotted accordingly. To compare the differences between the two groups, Student's *t*-test was used. *p* values of ≤ 0.05 were considered significant.

## 3 Results

### 3.1 Molecular docking of known B-RAF inhibitors

**Fig. 1:**
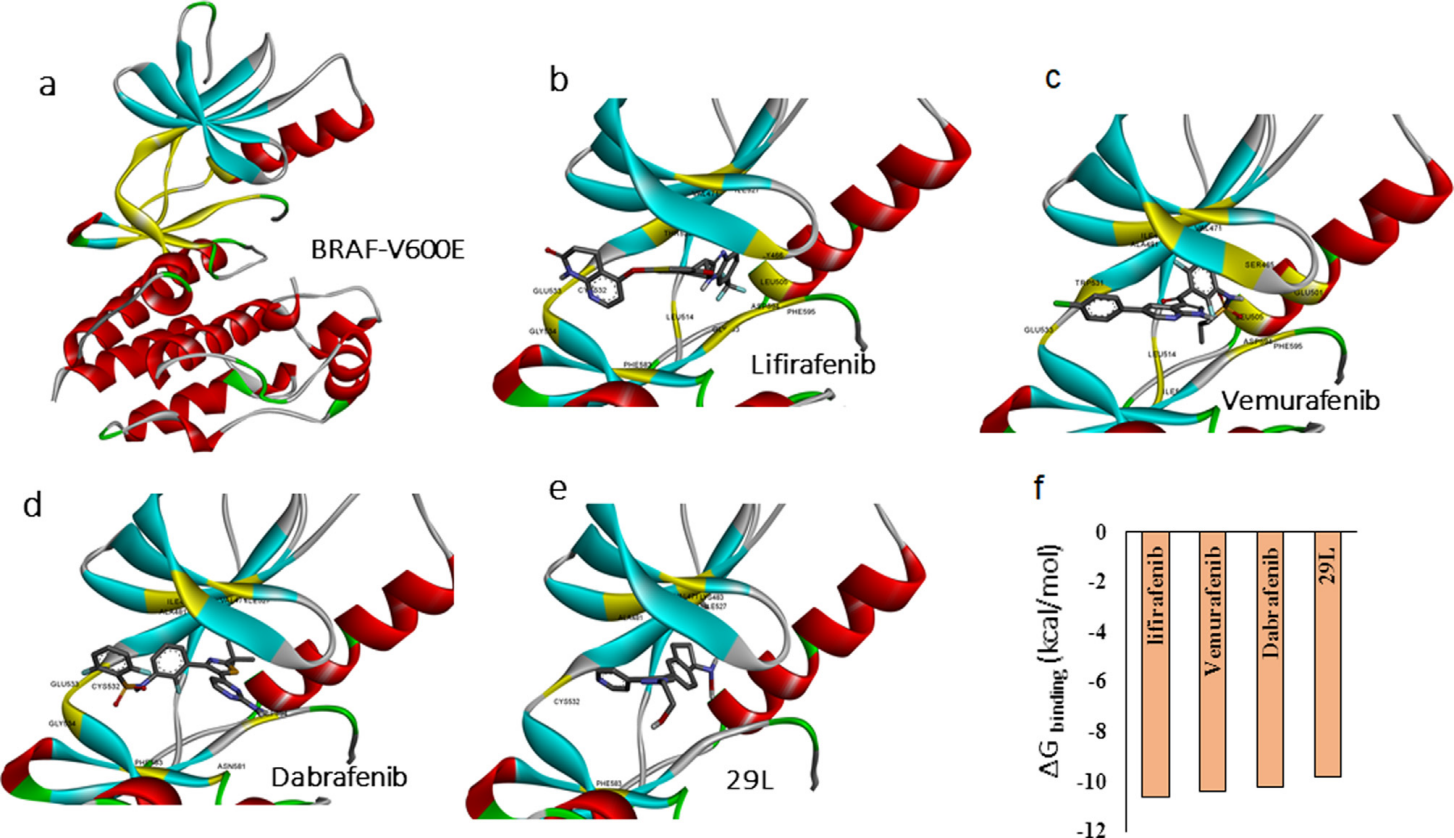
Protein-ligand interactions for known B-RAFV600E inhibitors. **(a)** Structure of B-RAFV600E, yellow highlighted region indicates the active site and ligand binding site. Predicted docking poses of known B-RAF inhibitors **(b)** Lifirafenib, **(c)** Vemurafenib **(d)** Dabrafenib and **(e)** 29L using Autodock VINA docking calculations. **(f)** Docking energy comparisons for the know inhibitors with B-RAF V600E.

In order to identify novel potent B-RAF-V600E inhibitors, we first determined the optimal docking conditions for the enzyme and ligand complex. We first performed protein–ligand docking of known B-RAF-V600E inhibitors. We chose four known B-RAF-V600E inhibitors: lifirafenib, vemurafenib, dabrafenib, and 29L. Docking calculations were based on the active site of the crystal structure of the B-RAF^-^V600E complex (PDBid: 4mnf) **(**Fig. 1**a)**. Docking analysis **(Fig b–e)** showed that lifirafenib and vemurafenib had the highest docking affinities with delta G binding energy of −11.7 and −10.7 kcal/mol, respectively, and compared to that of other known inhibitors **(**Fig. 1**e)**.

### 3.2 Top lead compounds against B-RAFV600E were identified by high-throughput virtual docking of the ChemBridge library compounds

**Fig. 2:**
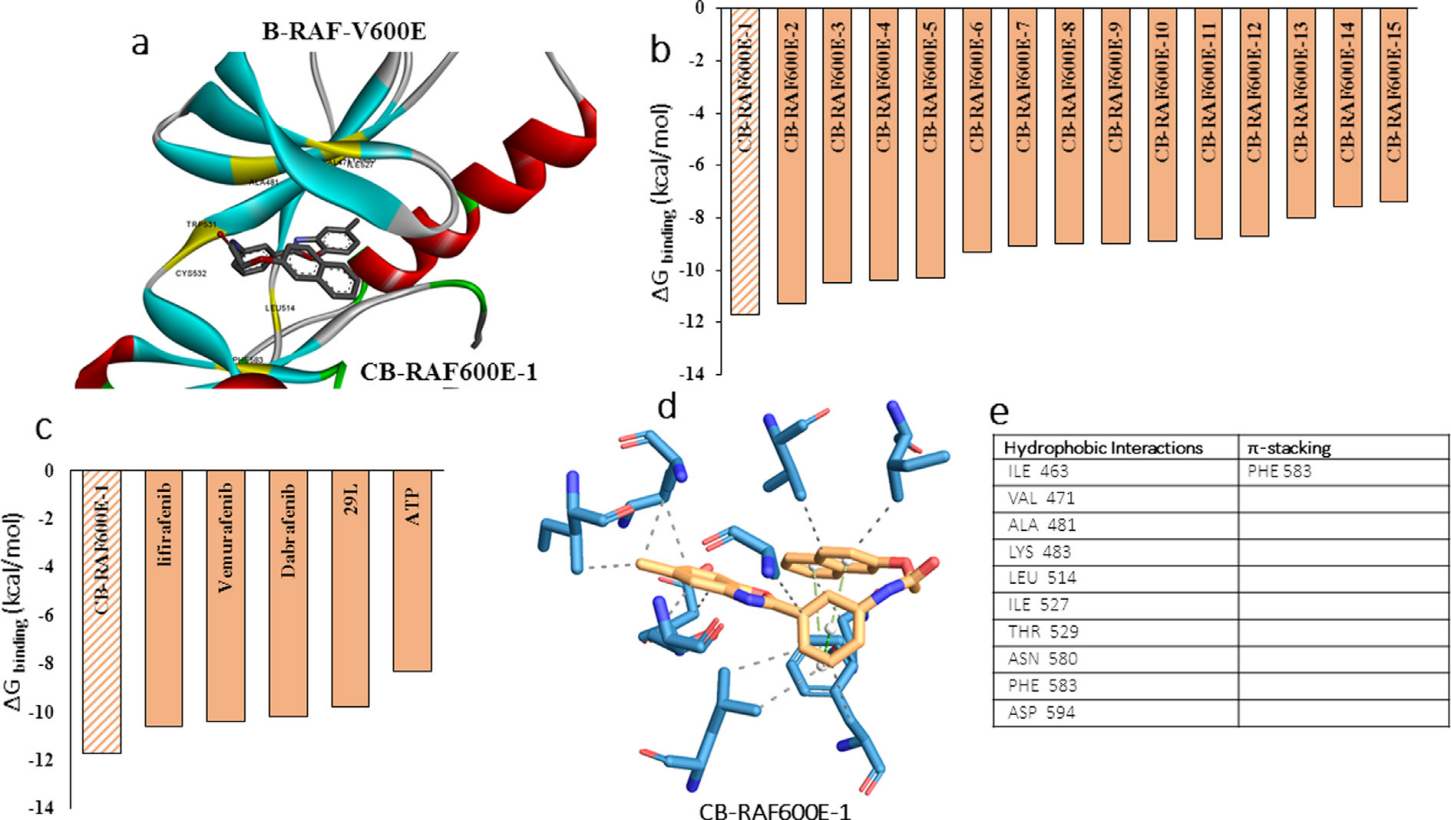
**(a)** Predicted binding pose of CB-RAF600E-1 with B-RAFV600E structure, yellow highlighted region indicates interacting residues of B-RAF with the ligand **(b)** Comparison of binding energies between top lead molecule CB-V600E-1 and other inhibitors (CB-RAF600E-2 to CB-RAF600E-15) from the library screened against B-RAFV600E. **(c)** Comparison of binding energies between top lead molecule CB-RAF600E-1 and known B-RAFV600E inhibitors. **(d)** PLIP analysis of protein–ligand interaction showing interacting amino acid residues of B-RAFV600E with CB-RAF600E-1 and **(e)** the type of interactions.

Based on our docking calculation results using known B-RAF-V600E inhibitors, we identified novel potent inhibitors from the ChemBridge small molecule library. We used KINAcore and KINAset, a library of kinase inhibitor-like small molecules from ChemBridge. The docking box was created based on known inhibitor docking calculations, as mentioned above. A total of 23,365 compounds were screened against the active site of the B-RAF-V600E structure, and the compounds were shortlisted based on their docking energies. The top 15 compounds from the high-throughput virtual docking were selected for stringent docking calculations to predict the best compound for experimental validation. We performed AutoDock VINA docking with a standard exhaustiveness of eight. While all the top 15 compounds bound to the active site (data not shown), CB-BrafV600E-1 emerged as the best lead compound with a docking score of ΔG_binding_ − 11.9 kcal/mol **(**Fig. 2 **a, b).** The binding energy of the compound was better than that of other known inhibitors **(**Fig. 2**c).** CB-BrafV600E-1 had multiple interactions with the B-RAF-V600E active site **(**Fig. 2**d)**. These include 10 hydrophobic interactions and a π-alkyl interaction, as summarized in the table **(**Fig. 2**e)**.

### 3.3 CB-RAF600E-1 predicted high binding energy towards Akt enzyme

**Fig. 3:**
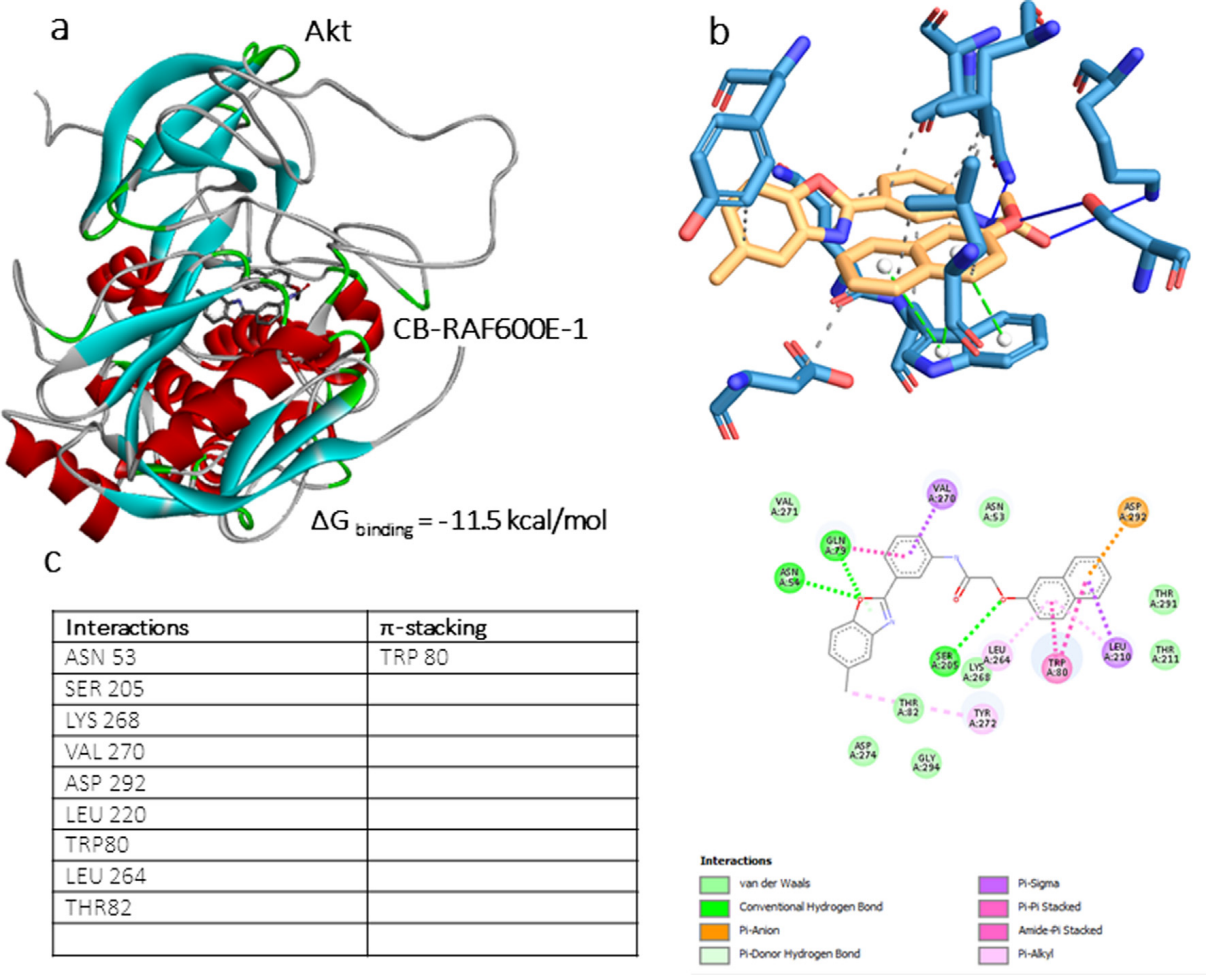
In silico docking of CB-RAF600E-1 to the crystal structure of Akt enzyme. **(a)** Full-length Akt enzyme crystal structure bound to the CB-RAF600E-1 with the binding energy indicated. **(b)** PLIP analysis for CB-RAF600E-1 associated ligand–protein interactions to Akt. **(c)** A 2-dimentional analysis of the protein–ligand amino acid interactions involved in CB-RAF600E-1 binding with Akt with the type of interactions involved.

To check whether CB-BRAFV600E could have an effect on the Pi3K/Akt pathway, computational docking with the Akt enzyme was performed. The known Akt inhibitor staurosporin was used to establish the docking position and grid box (data not shown). The compound had excellent binding prediction with the Akt enzyme with a ΔG_binding_ score of − 11.5 kcal/mol **(**Fig. 3**a)**. PLIP analysis revealed multiple interactions between CB-BrafV600E-1 and Akt **(**Fig. 3**b),** which included nine hydrophobic interactions and one π-alkyl interaction **(**Fig. 3**c)**.

### 3.4 CB-BrafV600E-1 inhibited B-RAFV600E and Akt kinases *in vitro*

**Fig. 4:**
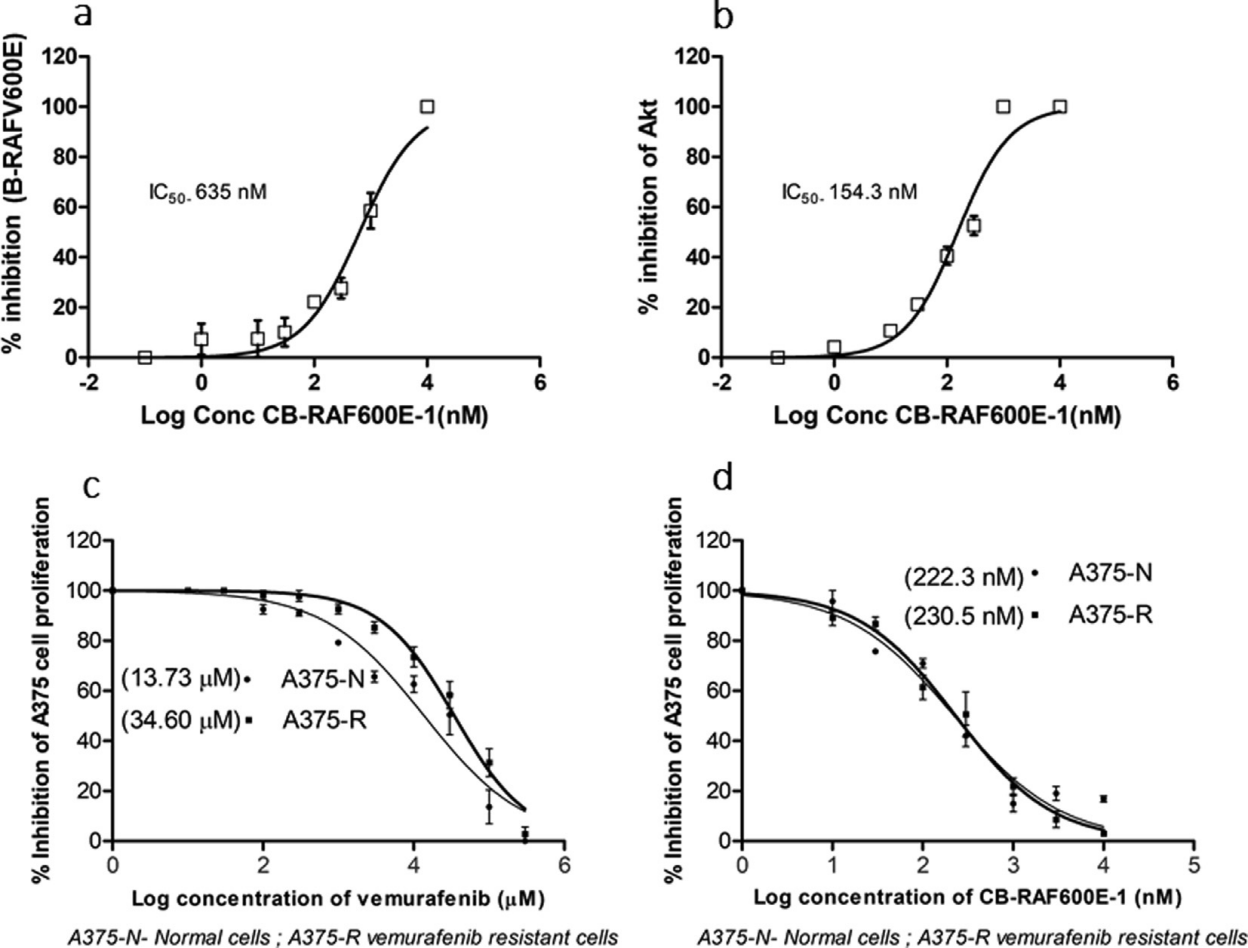
Enzyme IC_50_ values of CB-RAF600E-1 against **(a)** B-RAFV600E, **(b)** Akt. Assays were performed in respective ATP KM conditions. Results are expressed as mean ± SD from three separate experiments, and IC_50_ values are analyzed using GraphPad Prism version 6.0 software. **(c)** GI_50_ values for vemurafenib in 1 in normal and vemurafenib-resistant A375 cells. **(d)** GI_50_ values of CB-V600E-1 in normal and vemurafenib-resistant A375 cells. Cell proliferation was evaluated using MTT assay, and mean ± SD values of percentage cell inhibition were analyzed using GraphPad Prism version 6.0 software.

To further substantiate our computational predictions, we carried out *in vitro* enzyme inhibition studies for these enzyme targets. The compound dose-dependently inhibited B-RAFV600E with an IC_50_ value of 635 nM **(**Fig. 4**a)**. Similarly, a dose-dependent inhibitory effect was observed with the compound for Akt kinase with an IC_50_ value of 154.3 nM **(**Fig. 4**b).**

### 3.5 Efficacy of CB-BrafV600E-1 in normal and vemurafenib-resistant melanoma cells

Before examining the effect of CB-BrafV600E-1 in normal melanoma cells, we examined the effect of vemurafenib in normal and vemurafenib-resistant A375 cells. While vemurafenib had a GI_50_ value of 13.73 µM in normal A375 cells, the compound had a decreased effect with a GI_50_ value of 34.60 µM in the resistant A375 cells **(**Fig. 4**c)**. Testing the effect of CB-BrafV600E-1 in both normal and resistant melanoma cells did not alter the efficacy. The compound had 222.3 nM and 230.5 nM GI_50_ values in normal and vemurafenib-resistant cells, respectively **(**Fig. 4**d)**.

### 3.6 CB-BrafV600E-1 induced cell cycle changes and promoted apoptosis in normal and resistant types of melanoma cells

**Fig. 5:**
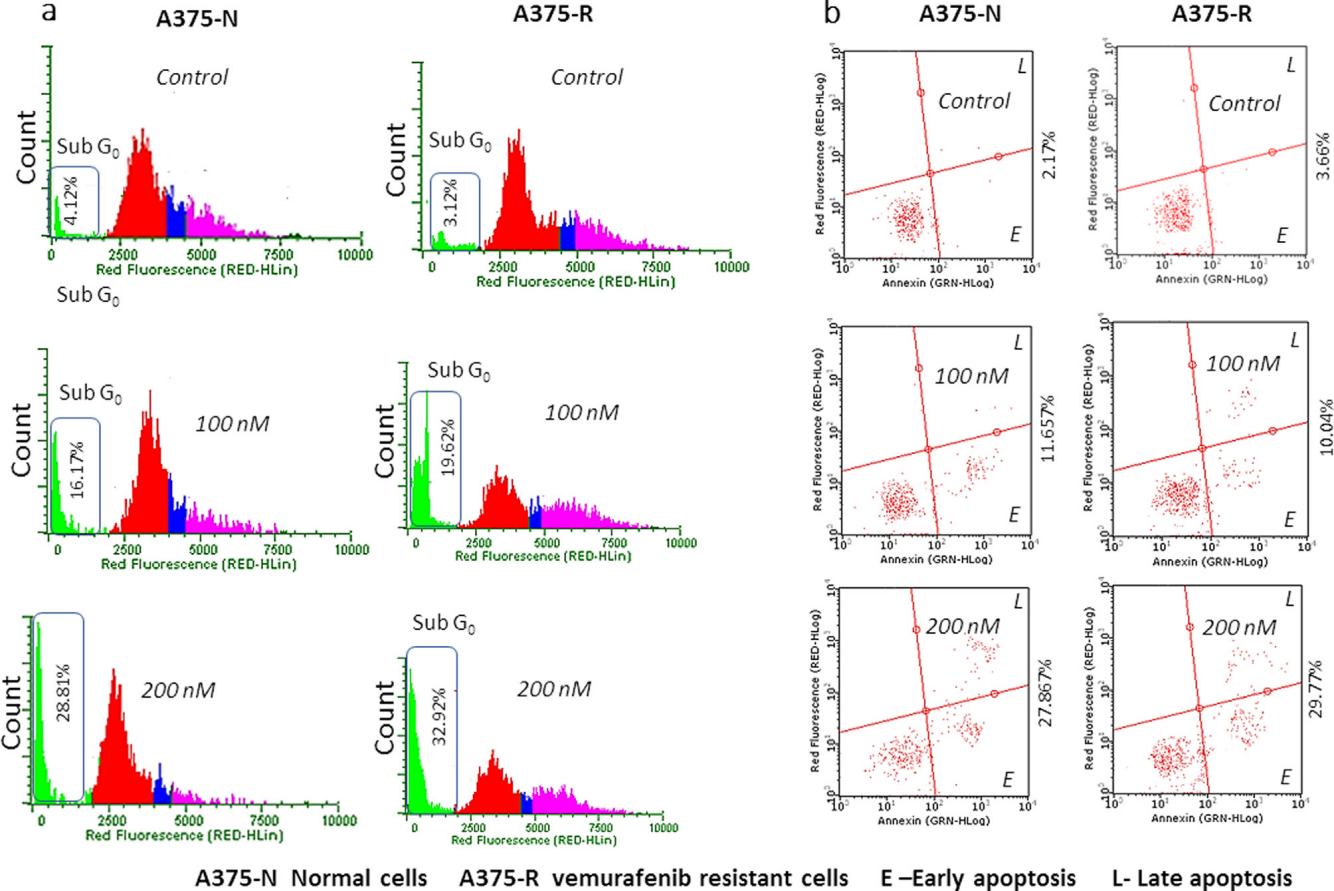
**(a)** Representative histograms from flow cytometry analysis of normal and vemurafenib-resistant A375 cell cycle with CB-RAF600E-1 treatments at the end of 72 h. Numerical values are mean ± SD percentage of sub G_0_/G_1_ phase cells from different experiments. **(b)** Annexin V staining indicating the early and late phase apoptotic cells in normal and vemurafenib-resistant A375 cells after CB-RAF600E-1 treatments. The compound increased early and late phase apoptotic populations for both these cells in a dose dependent way at 48 h. All experiments were performed thrice, and representative results are shown. Results are expressed as mean ± SD.

We next investigated whether the anti-proliferative efficacy of CB-BrafV600E-1 had other effects on the cellular functions of normal and resistant melanoma cells. When analyzed for the cell cycle of normal and resistant melanoma cells, we found that the compound had equal (potency) efficacy in both types of cells. Treatment of normal A375 cells with 100 nM of compound increased the sub G_0_/G_1_ population from 4.12% to 16.17% compared to the control **(**Fig. 5**a)**. A dose of 200 nM further increased this population to 28.81% **(**Fig. 5**a)**. Similarly, in the resistant cells, treatment with 100 nM compound showed 19.62% sub G_0_/G_1_ cells, and 200 nM treated cells showed 32.92% sub G_0_/G_1_ cells, with a corresponding 3.12% sub G_0_/G_1_ cells in untreated control cells **(**Fig. 5**a)**. When analyzed for apoptosis, treatment with CB-BrafV600E-1 increased the number of early and late apoptotic cells in both normal and resistant cell types, thereby increasing the total apoptosis in these cells **(**Fig. 5**b)**. Treatment with 100 nM and 200 nM compound increased the total apoptosis to 11.65% and 27.86% in normal cells, *respectively*, and 10.04% and 29.77% in resistant cells, respectively, while their respective controls had 2.17% and 3.66% total apoptotic cells, respectively, **(**Fig. 5**b)**.

### 3.7 Inhibition of key protein signaling in RAF/MEK/ERK and PI3K/Akt pathways

**Fig. 6:**
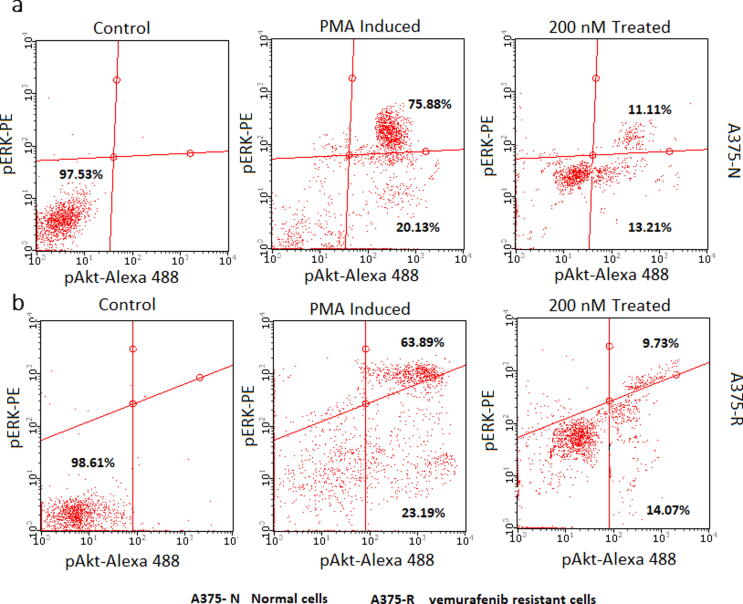
Flow cytometry enumeration of the percentage pERK/pAkt dual positive population in normal and vemurafenib-resistant A375 cells when induced with 50 ng/mL PMA for 5 min. The numerical values indicated are mean ± SD from different experiments. Pretreatment with 200 nM CB-RAF600E-1 caused a reduction in the pAkt/pERK positive population observed in both normal and resistant A375 cells.

To further showcase our *in vitro* enzyme assays and determine the mechanistic efficacy of the compound in cellular status, we carried out pERK and pAkt dual inhibition assays using flow cytometry. Induction with 50 ng/mL PMA for 5 min increased the pERK and pAkt dual-positive populations in both normal and vemurafenib-resistant A375 cells. The dual positive population in normal cells increased to 75.88% and that in resistant cells increased to 63.89% **(**Fig. 6**a, b)**. Treatment with CB-RAF600E-1 decreased the number of dual-positive cells to 11.11% in the normal cells and 9.73% in the resistant cells. PMA-induced control normal and resistant cells had 20.13% and 23.19% Akt single-positive cells, respectively **(**Fig. 6**a, b)**. CB-RAF600E-1 treatment reduced Akt single positivity to 13.21% and 14.07% in normal and resistant A375 cells, respectively **(**Fig. 6**a, b)**.

## 4 Discussion

Although represented by a small subset, melanoma continues to be a deadly cutaneous neoplasm, which is very common in youngsters compared to other cancer types (Whiteman et al., 2001). The RAS family is a class of small G-proteins responsible for MAPK signaling in downstream events. (Mercer and Pritchard, 2003, Dhomen and Marais, 2009). Mutated RAS is devoid of GTPase activity, which leads to uncontrolled cell proliferation and phenotype transformation in many cancer types, including melanoma (Marquette et al., 2007). RAS has also been shown to downregulate tumor suppressor genes, leading to aggravation of melanoma formation and worsening of the condition (Tsao et al., 2000). Given the involvement of RAS mutations in melanoma progression, this pathway has remained an active target in melanoma control for several years (Evans et al., 2013). Small molecules such as vemurafenib targeting B-RAFV600E have demonstrated clinical efficacy (Chapman et al., 2011). However, concerns regarding the efficacy of vemurafenib remain because of resistance and side effects such as rashes, fever, and joint pains (Flaherty et al., 2010). More seriously, patients who received vemurafenib treatment showed recurrence of the disease within 3 to 4 months of the drug response (Agarwala et al., 2010). It has been shown that in resistant cells, melanoma progresses through downstream signaling of the RAF and PI3K/Akt proteins (Dhomen and Marais, 2009), thus, we performed a high-throughput screening to identify a dual inhibitor against the V600E mutated B-RAF and Akt targets.

In this study, analysis of the interaction modes of known inhibitors with B-RAFV600E identified critical residues, which, at least in part, were responsible for their reported inhibitory activity. Our approach in computational docking fitted with the docking modes of known B-RAFV600E inhibitors. As an example, we used the 4MNF crystal structure, which was complexed with GDC-0879, a known B-RAFV600E inhibitor. When we docked the crystal structure with other known inhibitors such as lifirafenib, vemurafenib, dabrafenib, and 29L, the docking pattern similarly fitted in the same position for all these inhibitors, thereby confirming the active binding site of B-RAFV600E. Based on the high-throughput screening, followed by the standard docking protocol, CB-RAF600E-1 emerged as a potential lead compound with a binding pattern similar to that of known inhibitors, but with a greater binding energy. These results agreed with our *in vitro* findings of B-RAFV600E inhibition by CB-RAF600E-1, which was in the nanomolar range. Likewise, while the binding pattern of CB-RAF600E-1 was very similar to that of standard staurosporin against the Akt structure, the efficacy in terms of binding energy was better than that of staurosporin. The Akt binding efficacy of CB-RAF600E-1 was clearly (reflected) translated in the Akt enzyme inhibition assay. The compound was also effective in controlling the proliferation of normal melanoma cells.

Studies indicate that drug resistance can cause phosphorylated ERK-dependent paradoxical induction of proliferation in mutated B-RAF cells, thereby providing an alternative route for disease progression (Hatzivassiliou et al., 2010, Poulikakos et al., 2010). Our results were in accordance with these reports, as we observed a low efficacy of vemurafenib in the resistant A375 cells. On the other hand, CB-RAF600E-1 was equally effective in controlling both normal and vemurafenib-resistant melanoma cells, thereby suggesting that the dual inhibition efficacy of the compound was responsible for this activity. We demonstrated the dose-dependent efficacy of CB-RAF600E-1 in inducing apoptosis and altering the cell cycle in both normal and resistant melanoma cells. When analyzed for the mechanistic actions behind vemurafenib resistance, studies revealed that RAF dimerization via RAS activation along with over-activation of the MAPK pathway encoding ERK phosphorylation could be a possible reason (Johannessen et al., 2010, Solit and Rosen, 2011). In addition, B-RAF occurrence is likely to happen early and even to simultaneously alter the PI3K/Akt pathway for tumor progression. Therefore, targeted therapy directed towards both pathways would be more successful, with special reference to resistant melanoma cells (Goel et al., 2006). In this context, the efficacy of CB-RAF600E-1 in the enzyme inhibition assays of B-RAFV600E and Akt was also confirmed by the inhibition of phosphorylation of these proteins in normal and vemurafenib-resistant A375 cells, possibly explaining the reasons for the efficacy of the compound in both normal and resistant cell types.

## 5 Conclusion

In summary, we report that CB-RAF600E-1 is a potent, dual B-RAFV600E/Akt inhibitor, effective against both normal and vemurafenib-resistant melanoma cell lines. Further studies are recommended to develop this novel small molecule and its analogues to combat melanoma and its drug resistance.

## Funding

The author extends appreciation to the Deanship of Scientific Research at King Khalid University, Abha, Saudi Arabia, for funding this work through grant number R.G.P.1/228/42.

## Declaration of Competing Interest

The authors declare that they have no known competing financial interests or personal relationships that could have appeared to influence the work reported in this paper.
